# Patient drift and response-adaptive randomisation: impact and solutions

**DOI:** 10.1186/1745-6215-16-S2-P232

**Published:** 2015-11-16

**Authors:** Sofia S Villar, James Wason, Jack Bowden

**Affiliations:** 1 MRC Biostatistics Unit, Cambridge, UK

## 

There is a considerable interest in using response-adaptive randomisation in clinical trials. Several reasons motivate this. First, they improve individual patient response in clinical trials by increasing the allocation to a best performing treatment (if it exists). Skewing its allocation probability as data accumulates. Second, in multi-armed trials, by protecting the allocation to the control treatment, one can identify trial designs that score highly in both statistical power and patient benefit. Moreover, in this context, by testing many promising treatments simultaneously, multi-arm trials increase the probability of finding a successful new treatment, speeding up the process of doing so. Despite this, response-adaptive designs have seldom been used in practice. One of the major criticisms of response-adaptive randomisation is the potential for type-one error inflation due to “patient drift”. Patient drift can include changes in patients characteristics or the effectiveness of treatments over time. There have been few papers quantifying the extent to which this phenomena actually affects the operating characteristics and how to best correct for it.

In this talk we present work addressing both questions for novel adaptive randomisation methods. We consider several scenarios and allocation rules to assess how the type and magnitude of the drift affects the type-one error rate for these designs. We further suggest and assess statitiscal procedures that preserve type one error in the presence of unknown patient drift in both a two-arm and a multi-arm trial setting.

